# Editorial: T-Cell and Antibody-Mediated Rejection After Organ Transplantation in the Post-COVID-19 Era – Diagnosis, Immunological Risk Evaluation and Therapy

**DOI:** 10.3389/fimmu.2022.968668

**Published:** 2022-07-05

**Authors:** Antonij Slavcev, Caner Süsal, Ilias Doxiadis

**Affiliations:** ^1^ Department of Immunogenetics, Institute for Clinical and Experimental Medicine, Prague, Czechia; ^2^ Transplant Immunology Research Center of Excellence, Koç University Hospital, Istanbul, Turkey; ^3^ Institute for Transfusion Medicine, University Hospital, Leipzig, Germany

**Keywords:** T cell mediated rejection, antibody-mediated rejection, HLA, molecular microscope, COVID - 19

## Introduction

While organ transplantation centres around the world were coping with the severe consequences of the COVID-19 pandemic during the last years, immunological complications that occur after transplantation still represent an ever-growing medical and socio-economic problem. This Research Topic was envisioned to shed light on the growing application of new techniques and approaches, such as the molecular microscope and computational prediction of biomarkers, to improve rejection diagnosis and immunological risk evaluation in organ transplant recipients. Here, we briefly present the main highlights of the reports published in our topic.

Wahrmann et al. studied the influence on kidney transplant survival of Fc gamma receptor (FcγR) IIIA single nucleotide polymorphism FCGR3A-V/F158 in a large cohort of 1.940 donor/recipient pairs selected from the Collaborative Transplant Study (CTS) database. Analysing 10-year death-censored allograft survival, no differences in relation to *FCGR3A*-V/F158 variations were found. The study suggests that the previously reported association of the FCGR3A V/V-genotype with microcirculation inflammation (ref) does not play a role in kidney graft survival.

In the study of Trailin et al., laser capture microdissection combined with quantitative RT-PCR was used to discriminate the transcript patterns in kidney allografts of recipients at high risk of antibody-mediated rejection (AMR). The transcripts were either compartment-, AMR-, or “follow-up”-specific. The transcriptional profiles of early acute AMR shared similarities with ATN. The team also demonstrated distinct gene expression patterns in different renal compartments reflecting cellular infiltration.

The case study of Sherwood et al. investigated the immune profile of a kidney transplant recipient who encountered COVID-19 infection early post-transplant. Immunological monitoring of cellular, proteomic, and other parameters during the first 4 months post-infection was performed. The authors concluded that Basiliximab induction and maintenance triple immunosuppression did not impair the recipient’s ability to mount an immune response against COVID-19 detected during the first week post-transplant.

The team of Alfaro et al. searched for differentially expressed genes in patients with or without AMR, new target drugs, etc. and analysed their biological activities. The group reported an increase in the expression of genes related to various stages of immune activation, antigen presentation and antibody-mediated cytotoxicity during AMR. Computational approaches to look for new therapeutic uses of already allowed target drugs showed that the anti-cancer kinase inhibitor imatinib might be helpful in kidney transplantation for the prevention and/or treatment of AMR. These results have to be further confirmed for the eventual detection of highly expressed genes as rejection biomarkers/or as therapeutic targets to treat rejection in patients after kidney transplantation.

The aim of the group of Vietzen et al. was to investigate whether the *KLRC2* gene variants, which determine NKG2C expression, affect the pathogenicity of donor-specific antibodies (DSA) and influence long-term graft survival. They genotyped the KLRC2 wt/del variants for two distinct kidney transplant cohorts: (i) a cross-sectional cohort of 86 recipients who, on the basis of a positive post-transplant DSA result, all underwent allograft biopsies, and (ii) 1.860 recipients of a deceased donor renal allograft randomly selected from the CTS database. In the DSA-positive patient cohort, the *KLRC2* ^wt/wt^ variant was associated with AMR, microvascular inflammation, and elevated NK cell-related transcripts. However, neither in the DSA-positive nor in the CTS cohort, the *KLRC2* ^wt/wt^ allele was associated with impaired long-term allograft survival.

Baardwijk et al. developed and validated a transplant biopsy classifier using the genes of the Banff-Human Organ Transplant (B-HOT) panel obtained from the Molecular Microscope^®^ Diagnostic system (MMDx) microarray dataset. Gene expression data from almost 1.200 kidney transplant biopsies were used as training data for random forest models to predict kidney transplant biopsy Banff categories, including AMR, T-cell-mediated rejection and non-rejection. After performance assessment on cross-validation, the best-performing model was validated on an external independent dataset based on a different microarray version. The report demonstrates that, compared to the centralized and microarray-limited MMDx, a decentralized, open-access kidney transplant biopsy classifier can be developed that might work on data derived from different gene expression platforms and enable other centres to check and improve the tool. The multiplatform-compatible B-HOT panel proved to be a reliable predictor for classification of kidney transplant rejection.

## Author Contributions

AS wrote the editorial, CS critically reviewed the editorial, ID critically reviewed the editorial. All authors contributed to the article and approved the submitted version.

## Conflict of Interest

The authors declare that the research was conducted in the absence of any commercial or financial relationships that could be construed as a potential conflict of interest.

## Publisher’s Note

All claims expressed in this article are solely those of the authors and do not necessarily represent those of their affiliated organizations, or those of the publisher, the editors and the reviewers. Any product that may be evaluated in this article, or claim that may be made by its manufacturer, is not guaranteed or endorsed by the publisher.

